# Socio-Demographic, Clinical and Behavioral Characteristics Associated with a History of Suicide Attempts among Psychiatric Outpatients: A Case Control Study in a Northern Mexican City

**Published:** 2014-03

**Authors:** Cosme Alvarado-Esquivel, Luis Francisco Sánchez-Anguiano, Carlos Alberto Arnaud-Gil, Jesús Hernández-Tinoco, Luis Fernando Molina-Espinoza, Elizabeth Rábago-Sánchez

**Affiliations:** 1 Faculty of Medicine and Nutrition, Juárez University of Durango State, Durango, Mexico;; 2 Institute for Scientific Research, Juárez University of Durango State, Durango, Mexico;; 3 Hospital of Mental Health “Dr. Miguel Vallebueno”, Secretary of Health. Durango, Mexico

**Keywords:** Risk factors, Suicide attempts, Psychiatric patients, Mexico

## Abstract

**Background:**

Little is known about the epidemiology of suicide attempts among psychiatric outpatients in Mexico. This study was aimed to determine the socio-demographic, clinical and behavioral characteristics associated with suicide attempts in psychiatric outpatients in two public hospitals in Durango, Mexico.

**Methods:**

Two hundred seventy six psychiatric outpatients (154 suicide attempters and 122 patients without suicide attempt history) attended the two public hospitals in Durango City, Mexico were included in this study. Socio-demographic, clinical and behavioral characteristics were obtained retrospectively from all outpatients and compared in relation to the presence or absence of suicide attempt history.

**Results:**

Increased prevalence of suicide attempts was associated with mental and behavioral disorders due to psychoactive substance use (F10-19) (*P*=0.01), schizophrenia, schizotypal and delusional disorders (F20-29) (*P*=0.02), mood (affective) disorders (F30-39) (*P*<0.001), and disorders of adult personality and behavior (F60-69) (*P*<0.001). Multivariate analysis showed that suicide attempts were associated with young age (OR=1.21, 95% CI: 1.06-1.39; *P*=0.003), female gender (OR=2.98, 95% CI: 1.55-5.73; *P*=0.001), urban residence (OR=2.31, 95% CI: 1.17-4.57; *P*=0.01), memory impairment (OR=1.91, 95% CI: 1.07-3.40; *P*=0.02), alcohol consumption (OR=2.39, 95% CI: 1.21-4.70; *P*=0.01), and sexual promiscuity (OR=3.90, 95% CI: 1.74-8.77; *P*<0.001).

**Conclusions:**

We report the association of suicide attempts with socio-demographic, clinical and behavioral characteristics in psychiatric outpatients in Mexico. Results may be useful for an optimal planning of preventive measures against suicide attempts in psychiatric outpatients.

## INTRODUCTION

Suicidal behaviors represent a growing public health problem and a leading cause of injury and death worldwide (1, 2). Suicidal behaviors occur as frequent in developed as in developing countries (3). The cross-national lifetime prevalence of suicide attempts is 2.7% (4). However, the risk factors associated with suicide and suicide attempts vary substantially among countries (4-7) and ethnicity (1). In Mexico, a national epidemiology study revealed that suicide rate grew by 275% from 1970 to 2007 (8). This national study also showed that more than 6 million people had suicidal thoughts and nearly 10% of them attempted suicide (8). Therefore, suicide behaviors require urgent attention in Mexico. Research to reveal risk factors associated with suicide attempts including socio-demographic, clinical and behavioral factors is highly needed. Results from such epidemiological studies would be useful for an optimal design of preventive measures against suicide attempts. Some reported factors associated with increased risk for suicide attempts in Mexico include parental disorders (major depression, panic and anxiety disorders, substance dependence and antisocial personality disorder) (9), having a family background of alcoholism (10), parents’ divorce and family dysfunction (11), and gender (12). Since having a psychiatric diagnosis is a strong predictor for both fatal and non-fatal suicide attempt (13, 14), this group of population represents an important target population for studying the epidemiology of suicide attempts. However, very little is known about the characteristics of psychiatric patients who attempt suicide in Mexico. Therefore, this study aimed to determine the behavioral, clinical and socio-demographic characteristics associated with suicide attempts in psychiatric outpatients in a public psychiatric hospital in Durango, Mexico.

## METHODS

### Selection and description of participants

A case-control study was performed using data of 276 psychiatric outpatients previously obtained for a *Toxoplasma gondii* study (15) in Durango City, Mexico from September 2011 to June 2012. Of the 276 outpatients studied, 154 had suicide attempt history (cases) and 122 patients had no history of suicide attempts (controls). All 276 outpatients have attended the two public hospitals (Hospital of Mental Health “Miguel Vallebueno” and the General Hospital). Both hospitals belong to the same Secretary of Health and attend the same population; however, recent cases are most likely to be attended in the General Hospital but are sent to the other hospital for further psychiatric support. We used a non-probability (convenience) sampling. As a strategy to enroll participants, outpatients were invited to participate in the study when they were attending for consultations in the hospitals. Of the attended psychiatric patients, participants with suicide attempts outnumbered those without a history of suicide attempts and this fact contributed to have more cases than controls. However, we included as much patients as possible to obtain a representative sample. By the last months of sampling most attending outpatients had already been enrolled in the study and few new cases could be obtained. Only few patients (<5%) refused to participate. Inclusion criteria for the cases were psychiatric outpatients with history of suicide attempts, aged 18 years and older, regardless of gender, and psychiatric diagnosis. Cases included 117 females and 37 males aged 18-61 years old (mean 34.14+/-10.24 years). Inclusion criteria for controls were psychiatric outpatients without history of suicide attempts, aged 18 years and older, regardless of gender, and psychiatric disease. Controls included 74 females and 48 males aged 18-69 years old (mean 38.23+/-11.76 years). An exclusion criterion for cases and controls was missing any socio-demographic, clinical or behavioral data included in the study.

### Ethical aspects

Only archival data was used. Participation of outpatients in this study was voluntary. This study was approved by both the Ethical Committee of the Hospital of Mental Health “Dr. Miguel Vallebueno” of the Secretary of Health in Durango City and the Ethical Committee of the General Hospital of the Secretary of Health in Durango City. The purpose and procedures of the study were explained to all participants, and a written informed consent was obtained from each outpatient.

### Socio-demographic, behavioral and clinical characteristics of outpatients

Data of the socio-demographic, clinical and behavioral characteristics of the outpatients were obtained retrospectively from archival questionnaires. The questionnaires were filled by psychiatrics. The socio-demographic items asked about age, gender, residence, educational level, socio-economic status and occupation. Socio-economic status obtained was self-reported. Clinical data obtained from outpatients included the following: 1) psychiatric diagnosis, for this purpose the ICD-10 criteria (16) was used to classify the psychiatric diseases; 2) suicidal behavior, including history and number of suicide attempts, date of last suicide attempt, and method of suicide attempts. Diagnosis of suicide attempt was performed by psychiatrists. Suicide attempt was considered when a subject tried to kill him/herself. Self-harm without the clear intention to die was not considered suicide attempt; 3) general clinical characteristics as presence of any concomitant disease, frequent headache, impairment in memory, and history of transplant or surgery. Impairment in memory was self-reported. Behavioral data explored included alcohol consumption, drug abuse, sexual promiscuity, foreign travel, and raising pet animals (cats, dogs, birds).

### Statistics

Statistical analyses were conducted using the software Epi Info version 3.5.4 and SPSS 15.0 (SPSS Inc. Chicago, Illinois, USA). Difference in the frequencies between the groups was assessed using the Pearson’s chi-square test, or Fisher exact test or Yates’ correction when the assumptions for chi-square test were not met. Bivariate and multivariate analyses were used to evaluate the association between the characteristics of the outpatients and suicide attempts. Variables were included in the multivariate analysis if they had a *P* value equal to or less than 0.20 in the bivariate analysis. Age adjusted odd ratio (OR) and 95% confidence interval (CI) were calculated by multivariate analysis using multiple, unconditional, logistic regression. The criterion for statistical significance was set at *P* less than 0.05.

## RESULTS

Of the socio-demographic characteristics analyzed, suicide attempts were associated with age, gender, and residence area of the psychiatric outpatients in the bivariate analysis (Table 1). Higher prevalence of suicide attempts was observed in psychiatric patients aged 50 years old and younger, of female gender, and living in urban areas. Other socio-demographic characteristics in patients including educational level, occupation or socio-economic status did not show any significant association with suicide attempts.

**Table 1:** Socio-demographic characteristics of the patients and their relation with suicide attempts

Characteristic	No. of subjects tested	Subjects with suicide attempt		Odds ratio	95% Confidence interval	*P* value
		No.	%			

Age groups (years)						
30 or less	98	61	62.2	3.9	1.48-10.23	0.001
31-50	148	84	56.8	3.1	1.23-7.79	0.007
51-70	30	9	30	1.0		
Gender						
Male	85	37	43.5	1.0		
Female	191	117	61.3	2.1	1.18-3.56	0.006
Residence place						
Durango State	265	146	55.1	1.0		
Other Mexican State	11	8	72.7	2.2	0.51-10.59	0.35
Residence area						
Urban	221	132	59.7	4.2	1.33-13.74	0.004
Suburban	36	17	47.2	2.5	0.65-10.11	0.13
Rural	19	5	26.3	1.0		
Educational level						
No education	4	3	75	3.2	0.27-86.60	0.61
1 to 6 years	65	32	49.2	1.1	0.47-2.32	0.9
7-12 years	155	94	60.6	1.7	0.84-3.29	0.11
>12 years	52	25	48.1	1.0		
Occupation						
Agriculture or livestock	11	4	36.4	1.1	0.15-8.85	1
Business	28	14	50	2.0	0.40-10.39	0.33
Clerck	56	38	67.9	4.2	0.97-19.60	0.05
Construction or factory	7	3	42.9	1.5	0.15-15.62	1
Housekeeping	109	62	56.9	2.6	0.66-11.19	0.11
None	39	19	48.7	1.9	0.42-9.14	0.34
Professional	12	4	33.3	1.0		
Student	14	10	71.4	5.0	0.73-39.29	0.05
Socio-economic level^a^						
Low	102	56	54.9	1.0		
Medium	168	94	56	1.0	0.62-1.76	0.86
High	6	4	66.7	1.6	0.24-13.60	0.69

a Self-reported.

The psychiatric diagnoses of psychiatric outpatients and their associations with suicide attempts are shown in Table 2. Higher prevalence of suicide attempts was associated with the following psychiatric diagnoses: mental and behavioral disorders due to psychoactive substance use (F10-19) (*P*=0.01), schizophrenia, schizotypal and delusional disorders (F20-29) (*P*=0.02), mood (affective) disorders (F30-39) (*P*<0.001), and disorders of adult personality and behavior (F60-69) (*P*<0.001). In contrast, the lowest prevalence of suicide attempts was observed in patients suffering from neurotic, stress-related and somatoform disorders (F40-49).

**Table 2:** Psychiatric diagnoses of the patients studied and their relation with suicide attempts

Code	Diagnosis	No. of subjects tested	Subjects with suicide attempt		Odds ratio	95% Confidence interval	*P* value
			No.	%			

F00-09	Organic, including symptomatic, mental disorders	16	5	31.3	1.8	0.42-7.80	0.48
F10-19	Mental and behavioral disorders due to psychoactive substance use	16	9	56.3	5.1	1.29-21.31	0.01
F20-29	Schizophrenia, schizotypal and delusional disorders	24	11	45.8	3.4	1.01-11.61	0.02
F30-39	Mood (affective) disorders	149	101	67.8	8.4	3.54-20.54	<0.001
F40-49	Neurotic, stress-related and somatoform disorders	45	9	20	1.0		
F60-69	Disorders of adult personality and behavior	17	14	82.4	18.7	3.78-105.81	<0.001
F70-79	Mental retardation	9	5	55.6	5.0	0.90-29.21	0.07

Of the 154 suicide attempters studied, 138 (89.6%) patients had had from 1 to 5 suicide attempts, 14 (9.1%) patients had had from 6 to 10 suicide attempts, and 2 (1.3%) patients had had more than 11 suicide attempts. The last suicide attempt in outpatients had occurred from few days to more than 5 years ago (73 had <6 months, 23 between 6-11 months, 28 between 1-5 years, and 30 >5 years). Eleven different methods for suicide attempts were reported by the patients being medicament consumption the most frequently used (129, 83.8%), followed by wrist cutting (39, 25.3%), hanging (31, 20.1%), vehicular impact (5, 3.2%), pesticide poisoning (3, 1.9%), gas inhalation (3, 1.9%), jumping from height (1, 0.6%), drug overdose (1, 0.6%), head hits (1, 0.6%), alcohol poisoning (1, 0.6%), and fertilizer poisoning (1, 0.6%). Medications used for suicide attempts included benzodiazepines, aspirin, diclofenac and paracetamol.

With respect to general clinical characteristics, higher proportion of suicide attempters had some form of memory impairment than patients without suicide attempts (*P*=0.005) (Table 3). Other general clinical characteristics in outpatients including presence of any concomitant disease, frequent headache, and history of transplant or surgery were observed in a similar frequency in suicide attempters and patients without history of suicide attempts. Of the behavioral characteristics explored (Table 3), suicide attempters had a significantly higher prevalence of alcohol consumption and sexual promiscuity than outpatients without suicide attempts (*P*=0.001 and *P*<0.001, respectively). Other behavioral characteristics including drug abuse, foreign travel, and raising pet animals were not associated with suicide attempts by bivariate analysis.

**Table 3:** Bivariate analysis of clinical and behavioral data in suicide attempters and controls

Characteristic	Suicide attempters (n=154)		Control subjects (n=122)		*P* value
	No. of subjects	%	No. of subjects	%	

Underlying disease					
Yes	43	27.9	39	32	0.46
No	111	72.1	83	68	
Headaches frequently					
Yes	106	68.8	76	62.3	0.25
No	48	31.2	46	37.7	
Memory impairment					
Yes	113	73.4	70	57.4	0.005
No	41	26.6	52	42.6	
Transplantation					
Yes	1	0.6	1	0.8	1
No	153	99.4	121	99.2	
Surgery ever					
Yes	82	53.3	62	50.8	0.68
No	72	46.8	60	49.2	
Alcohol consumption					
Yes	66	42.9	29	23.8	0.001
No	88	57.1	93	76.2	
Drug abuse					
Yes	31	20.1	15	12.3	0.08
No	123	79.9	107	87.7	
Sexual promiscuity					
Yes	42	27.3	12	9.8	<0.001
No	112	72.7	110	90.2	
Raising pet animals					
Yes	104	67.5	73	59.8	0.18
No	50	32.5	49	40.2	
Traveling abroad					
Yes	44	28.6	37	30.3	0.75
No	110	71.4	85	69.7	
National trips					
Yes	123	79.9	98	80.3	0.97
No	31	20.1	24	19.7	

Logistic regression analysis of socio-demographic, clinical and behavioral variables with a *P* value equal to or less than 0.20 (Table 4) showed that suicide attempts were associated with age (OR=1.21, 95% CI: 1.06-1.39; *P*=0.003), female gender (OR=2.98, 95% CI: 1.55-5.73; *P*=0.001), urban residence (OR=2.31, 95% CI: 1.17-4.57; *P*=0.01), memory impairment (OR=1.91, 95% CI: 1.07-3.40; *P*=0.02), alcohol consumption (OR=2.39, 95% CI: 1.21-4.70; *P*=0.01), and sexual promiscuity (OR=3.90, 95% CI: 1.74-8.77; *P*<0.001). Other socio-demographical, clinical and behavioral characteristics including, education, occupation, presence of underlying disease, drug abuse and raising pet animals were not associated with suicide attempts through multivariate analysis.

**Table 4:** Multivariate analysis of selected characteristics of participants and their association with suicide attempts

Characteristic	Odd ratio	95 % confidence interval	*P* value

Age	1.21	1.06-1.39	0.003
Female sex	2.98	1.55-5.73	0.001
Urban residence	2.31	1.17-4.57	0.01
Education	1.14	0.75-1.73	0.51
Occupation	1.09	0.97-1.23	0.13
Memory impairment	1.91	1.07-3.40	0.02
Alcohol consumption	2.39	1.21-4.70	0.01
Drug abuse	1.10	0.46-2.64	0.81
Sexual promiscuity	3.90	1.74-8.77	<0.001
Raising pet animals	1.42	0.81-2.48	0.21

## DISCUSSION

In the present study a number of epidemiological characteristics in psychiatric outpatients associated with suicide attempts were found. Multivariate analysis showed that suicide attempts were associated with young age, female gender, urban residence, memory impairment, alcohol consumption, and sexual promiscuity. Age has been associated with suicide attempts in other studies in Mexico and other countries. In a cross-sectional survey in patients of a hospital in Mexico City, researchers found that age was associated with suicide attempts (17). In cross-national studies, nonfatal suicide behaviors were more prevalent in persons who were young (1, 3). Similarly, female gender has been associated with suicide attempts in other studies. In a Turkish study, researchers found that the rate of suicides attempts was highest in women (6). In a cross-national study in 21 countries, Borges *et al*. (3) found that female sex was a risk factor for suicidal behavior in both developed and developing countries. It is not clear why the frequency of suicide attempts was higher in young and female patients than old and male patients. It is possible that differences in risk factors for suicide attempts existed among subgroups. Major depression is more frequent in women than in men (18) and the higher rates of depression are seen among women of reproductive age (19) such factors might have contributed for the increased frequency of suicide attempts in the female patients studied. With respect to the association of urban residence with suicide attempts found in the present study, such result agrees with that reported in a study in the United Kingdom. Higher rates of urban deliberate self-harm rates were higher than rural rates amongst both males and females aged 15 years and over presenting to the general hospital in Oxfordshire, England (20). In contrast, the result in the present study conflicts with that reported in a prospective population-based study of suicidal behaviors by burns requiring hospitalizations in a province in Iran, where the rate of suicidal behavior by burns among the rural population was significantly higher than the urban population (21). Differences in the characteristics of the patients among the studies may explain the differences in the associations with urban or rural residence including methods for suicide attempts and psychiatric diagnosis. Concerning the association of suicide attempts with alcohol consumption found in the present study, such result confirms previous observations. Alcoholism had been associated with a high risk of suicidal behavior (22). In a survey in patients in a general hospital in Mexico City, high levels of alcohol consumption was associated with suicide attempts (17). In a study in bipolar disorder patients in the USA, a history of alcohol abuse was associated with greater probability of a suicide attempt (23). Remarkably, in the current study an association of suicide attempts with sexual promiscuity was found. To our knowledge this association has not been reported in the medical literature. Little is known about the relation of suicide attempts with high or low sexual activity in psychiatric patients. In a study of patients with bipolar disorder in Pisa, Italy, lifetime sexual dysfunctions were associated with lifetime suicide attempts (24). It is not clear why suicide attempts were associated with sexual promiscuity in the present study. It is possible that such association might be related with hormones in patients. Testosterone levels have been positively correlated with the number of suicide attempts in patients with bipolar disorder (25). On the other hand, in a National Longitudinal Study of Adolescent Health in the USA, researchers found that sexual activity was associated with significantly increased odds of depression, suicidal ideation, and suicide attempts (26). In addition, girls with sexual activity were found to experience more depressive symptoms than boys with similar behavior (27).

Psychiatric patients have a higher risk for suicide attempts than the general population (28). In fact, in the psychiatric outpatients studied suicide attempters outnumber patients without suicide attempts. The prevalence of suicide attempts was particularly high in outpatients with mood (affective) disorders, schizophrenia, schizotypal and delusional disorders, disorders of adult personality and behavior, and mental and behavioral disorders due to psychoactive substance use. The high rates of depression and alcohol consumption in such patients might have contributed for suicide attempts. Our results agree with those reported in other studies. In an observational study of patients admitted to a university hospital in Spain, the risk of suicide attempts was highest in patients suffering from mood or affective disorders and personality disorders (29). In a meta-analysis of 21 studies, depressive disorders and borderline personality disorders were also associated with non-suicidal self-injury (30).

Of note, suicide attempters had a significantly higher frequency of memory impairment than patients without suicide attempts. This result is consistent with those reported in a recent study, where verbal working memory performance was reduced in suicide attempters compared to patients with a history of mood disorders but with no history of suicidal acts and healthy controls with no history of mood disorders or suicide attempts (31). In another study, women with borderline personality disorders and suicide attempts showed reduce specificity of autobiographical memory as compared with controls (32). In addition, in a survey of suicide attempters over 60 years of age admitted to a psychiatric hospital in France, female patients with memory disorders showed an increased risk of repeated suicide attempt (33). In contrast, in a study of persons 70 years and older who sought hospital treatment after a suicide attempt, no association of suicide attempts with dementia was found (34). In a recent study, researchers found that neurocognitive vulnerability to suicidal behavior may rely on impairments in value-based decision making and cognitive control (35).

The present study has some limitations. Since we used archival data obtained for a seroepidemiology study, some important information related with suicide attempts including marital status, living alone, and others were not obtained. We did not match cases and controls by age or gender. The number of patients was also limited and we were unable to find as many controls as cases. These facts do not allow generalizing our results. Further research with larger sample sizes of psychiatric patients is needed.

## CONCLUSIONS

We report the association of suicide attempts with socio-demographic, clinical and behavioral characteristics in psychiatric outpatients in Mexico. Female gender, young age, urban residence, type of psychiatric disease, memory impairment, alcoholism and sexual promiscuity are important factors associated with suicide attempts. Results may be useful for an optimal planning of preventive measures against suicide attempts in psychiatric outpatients. Further research on the link of suicide attempts with sexual behavior, substance dependence and cognition in psychiatric patients should be conducted.
